# Dr. G. S. West

**Published:** 1905-01

**Authors:** 


Dr. G. S. West.—Died at his home in Palestine, Texas, December 27th (ult.), Dr. G. S. West, aged 81. Dr. West was one of the most distinguished surgeons in the Confederate army, and was the first one to whom a commission was issued by the war department. Dr. West graduated from the Medical Department of the University of New York in 1854. When yellow fever broke out with great violence in Norfolk, Virginia, in 1856, he went from his home in New York to the stricken people and gave his services freely to all classes. After the epidemic was over the citizens were so pleased with him and so grateful that they gave him a residence and guaranteed him $3000 a year to remain amongst them. He accepted it. When the war broke out they said to him, "We know you wish to go North and go in the Union army; do so. After the war, return to us, and live with us." Dr. West said, "I cast my lot with the South when I came here. I will enter the Southern army." He did so, and filled many positions of honor and usefulness. During those last terrible days around Petersburg and Richmond he was chief of the operating staff in the field. I was on duty with him in the hospitals in Georgia in 1864, and knew him well. Our acquaintance and friendship remained unbroken up to the day of his death, and I rarely ever met a man who possessed as he did so many excellent and lovable qualities. In his latter years he became very deaf,—due to the cannonading about him at the downfall of the Southern Capital and Petersburg,—and while able to make a living he underwent many privations. His only child—a son—died after reaching man's estate. His devoted wife is his sole survivor. Peace to his ashes; and may that peace that passeth all understanding be with his bereaved helpmate and companion through all his struggles and adversity.

